# Distressing dreams and risk of Parkinson's disease: A population-based cohort study

**DOI:** 10.1016/j.eclinm.2022.101474

**Published:** 2022-06-08

**Authors:** Dr Abidemi I. Otaiku

**Affiliations:** a Department of Neurology, Birmingham City Hospital, Birmingham, UK; b Centre for Human Brain Health, University of Birmingham, Birmingham, UK

**Keywords:** Dreaming, Parkinson's disease, Nightmares

## Abstract

**Background:**

Parkinson's disease (PD) is associated with alterations to the phenomenology of dreaming - including an increased frequency of distressing dreams. Whether distressing dreams may precede the development of PD is unknown. This study investigated the association between frequent distressing dreams and the risk of incident PD.

**Methods:**

3818 men aged 67 years or older from the Osteoporotic Fractures in Men Study (MrOS), a population-based cohort from the USA, who were free from PD at baseline (December 2003 – April 2011) and completed item 5h of the Pittsburgh Sleep Quality Index - which probes the frequency of distressing dreams in the past month, were included in this analysis. Incident PD was based on doctor diagnosis. Multivariable logistic regression was used to estimate odds ratios (OR) for incident PD according to distressing dream frequency, with adjustment for potential confounders.

**Findings:**

During a mean follow-up of 7·3 years, 91 (2·4%) cases of incident PD were identified. Participants with frequent distressing dreams at baseline had a 2-fold risk for incident PD (OR, 2·01; 95% CI, 1·1-3·6, *P* = 0.02). When stratified by follow-up time, frequent distressing dreams were associated with a greater than 3-fold risk for incident PD during the first 5 years after baseline (OR, 3·38; 95% CI, 1·3-8·7; *P* = 0·01), however no effect was found during the subsequent 7 years (OR, 1·55; 95% CI, 0·7-3·3; *P* = 0·26).

**Interpretation:**

In this prospective cohort, frequent distressing dreams were associated with an increased risk for incident PD. The association was only significant within the 5 years prior to diagnosis, which suggests that frequent distressing dreams may be a prodromal symptom of PD.

**Funding:**

The study received no external funding.

**Research in context**

**Evidence before the study**

A PubMed search was conducted using the search string: ((‘nightmares’ OR ‘bad dreams’ OR ‘vivid dreams’ OR ‘dream content’) AND (‘Parkinson's disease’)); to identify primary research studies published in any language up until April 21, 2022. Cross-sectional studies have shown that people with Parkinson's disease (PD) experience nightmares and bad dreams more frequently than adults in the general population. However, no prospective study has evaluated whether frequent distressing dreams (i.e., nightmares or bad dreams) in community-dwelling adults, may be associated with an increased risk for developing PD.

**Added value of this study**

This population-based prospective study has shown for the first time that frequent distressing dreams (≥1/week) in community-dwelling older adults, may be associated with an increased risk for developing PD. In this study, the risk conferred by having frequent distressing dreams was greater than 3-fold within the 5 years preceding a PD diagnosis. These findings open the door for future studies on the association between distressing dreams and the subsequent development of PD, especially amongst women and younger adults.

**Implications of all the available evidence**

Frequent distressing dreams in older men are associated with an increased risk for developing PD and may represent prodromal symptoms. Screening for late onset distressing dreams may help to identify individuals at risk of developing PD.

Alt-text: Unlabelled box

## Introduction

Parkinson's disease (PD) is the second most common neurodegenerative disease and the fastest growing neurological disorder in the world.^1^ As a chronic and progressive condition, with a disease duration that can span decades, PD often leads to significant disability for affected individuals and negatively impacts on quality of life.^2^ PD also has a profound effect on caregivers,^2^ as well as a high total economic burden.^3^

With the ageing of the world's population, decline in smoking rates, and changes in environmental risk factors linked to PD, it is projected that the global prevalence of PD could double within the next decade.^1^ Given that PD remains incurable, identifying individuals who are at high risk for developing PD - in whom interventions could be targeted, is now considered an urgent priority.

In recent years, it has been shown that people with PD experience a wide range of non-motor problems (including depression, constipation, urinary dysfunction and excessive daytime sleepiness) alongside the characteristic motor features of PD.^2^ Moreover, it is well established that individuals in the general population who have these issues, may be at significantly higher risk of developing PD in the future.^4^

Intriguingly, cross-sectional studies have shown that PD is also associated with alterations to the phenomenology of dreaming.^5^ This includes changes to dream content (increased negative emotions, increased physical aggression and higher prevalence of animal characters),^6^ as well as the development of rapid eye movement sleep behaviour disorder (RBD) – or “dream enactment behaviours” (DEBs).^5^ In addition, people with PD are approximately four times more likely than adults in the general population, to experience nightmares sufficiently frequent to be considered a clinical disorder.^7^

Although recent research has shown that a quarter of patients with PD may experience frequent distressing dreams (nightmares or bad dreams) from the time of diagnosis,^8^^,^^9^ and that these patients may be at higher risk for early motor and cognitive decline;^9^ it remains to be investigated whether frequent distressing dreams in the general population may be associated with the risk of developing PD.

This study investigated the hypothesis that frequent distressing dreams would be associated with an increased risk for developing PD, and tested this association using data from the Osteoporotic Fractures in Men (MrOS) study,^10^ with up to 12-years follow-up for incident PD.

## Methods

### Study design and participants

MrOS is an observational, longitudinal cohort study that enrolled 5994 community-dwelling men aged 65 years or older at six clinical centres in the USA, including: Birmingham, Minneapolis, Palo Alto, Pittsburgh, Portland, and San Diego.^10^ The baseline assessments were conducted between March 2000 – April 2002 (Visit 1).

The present analysis used data from: Sleep Visit 1 (December 2003 – March 2005), Visit 2 (March 2005 – May 2006), Visit 3 (March 2007 – March 2009), Interim 2 (March 2009 – April 2011), Sleep Visit 2 (November 2009 – March 2012), and Visit 4 (May 2014 – May 2016). Sleep Visit 1 and Interim 2 were considered as the baseline for this study, since these were the earliest visits where information on distressing dreams and history of PD were assessed. Interim 2 was considered baseline for the men that were not included in Sleep Visit 1.

To be included in this analysis, participants must have been free from PD at baseline and have completed the questionnaire item on distressing dreams (*n* = 4595). Of these participants, those with missing data for any covariates included in the primary analysis (*n* = 70) or who had died (*n* = 487) or ended participation before follow-up (*n* = 220), were excluded. This yielded an analytic sample comprising 3818 men, including 3006 (78·7%) men from Sleep Visit 1 and 812 (21·3%) men from Interim 2.

### Distressing dreams

Participants completed the Pittsburgh Sleep Quality Index (PSQI) at baseline, a validated self-report questionnaire for assessing subjective sleep quality and disturbances.^11^ Distressing dreams were assessed using item 5h of the PSQI: “During the past month, how often have you had trouble sleeping because you have bad dreams?”. No definition of bad dreams was provided. The response options included: (0) “not during the past month, (1) “Less than once a week”, (2) “Once or twice a week” and (3) “Three or more times a week”. Participants experiencing distressing dreams at least once a week were considered to have frequent distressing dreams, consistent with previous studies.^12^

### Ascertainment of incident PD

During the 12-year follow-up, participants were asked at each clinical visit or questionnaire-based contact, to report whether they had ever been diagnosed with PD by a doctor. Incident PD was defined as doctor-diagnosed PD. Follow-up time was calculated for each participant as the time to the most recent collection of data on PD diagnosis.

### Covariates

Potential confounders were chosen based on *a priori* knowledge of factors associated with nightmares and PD risk, as well as previous studies.^12^, ^13^, ^14^, ^15^ These included: age in years (continuous), ethnicity (white, black, Asian, other, mixed), educational qualifications (college degree, high school degree, none), smoking status (current, past, never), alcohol intake (drinks/week, <1, 1-13, ≥14), current depression (yes/no), history of doctor-diagnosed diabetes mellitus (yes/no), history of doctor-diagnosed hypertension (yes/no), sleep onset insomnia (times/week, 0, <1, 1-2, ≥3), sleep maintenance insomnia (times/week, 0, <1, 1-2, ≥3), self-rated health (good/excellent, poor/fair), physical activity levels (continuous), and use of medications associated with dream content changes (yes/no). In addition, current anxiety (yes/no), excessive daytime sleepiness (yes/no), global cognitive function (normal cognition, mild-severe cognitive impairment) and severity of sleep-disordered breathing (apnoea-hypopnea index [AHI], <15, ≥15), were available for the participants that took part in Sleep Visit 1.

Age, ethnicity, educational qualifications, alcohol intake, smoking status, history of doctor-diagnosed diabetes mellitus, history of doctor-diagnosed hypertension, and self-rated health, were self-reported at baseline. Depressive symptoms were evaluated using the Geriatric Depression Scale, with scores ≥6 indicating clinically significant depression.^16^ Sleep onset insomnia and sleep maintenance insomnia were assessed using Item 5a and 5b of the PSQI respectively (scores ranging from 0 “not during the past month”, to 3 “three or more times a week”).^11^ Level of physical activity was estimated using the Physical Activity Scale for the Elderly (scores range from 0-793, with higher scores indicating greater physical activity levels).^17^ Anxiety symptoms were evaluated using the Goldberg Anxiety Scale, with scores ≥5 indicating clinically significant anxiety.^18^ Global cognitive function was measured using The Modified Mini-Mental State Examination, with scores ≤88 indicating mild-severe cognitive impairment.^19^ Daytime sleepiness was assessed using The Epworth Sleepiness Scale, with scores ≥11 indicating excessive daytime sleepiness.^20^ Sleep-disordered breathing was assessed using polysomnography and defined with the AHI (apnoeas or hypopneas per hour of sleep with a desaturation of ≥3%) as previously described.^21^ At each visit, participants were asked to bring in all the medications they had used in the last 30 days. Medication use was defined as: antidepressants, benzodiazepines, beta blockers, alpha blockers, antipsychotics, amphetamines, cholinesterase inhibitors, melatonin, and nonbenzodiazepine, nonbarbiturate sedative hypnotics.^5^^,^^22^

### Statistical analysis

Characteristics of the participants at baseline were compared using χ^2^ tests for categorical variables, independent samples t-tests for normally distributed continuous variables, and Mann–Whitney U tests for nonnormally distributed variables.

Multivariable logistic regression was used to obtain odds ratios (ORs) and 95% confidence intervals (CIs) to assess the longitudinal association between frequent distressing dreams and risk of PD with adjustment for potential confounders. Model 1 was minimally adjusted for age and clinic site. Model 2 additionally adjusted for race, education, smoking status, alcohol intake, depression, sleep onset insomnia, sleep maintenance insomnia, diabetes, hypertension, physical activity levels, self-rated health and medication use.

To explore the temporal relationship between distressing dreams and PD, a stratified analysis was carried out using different time-periods: baseline to < 5·5 years, and 5·5 to 12 years. As a sensitivity analysis, the primary analysis was repeated after further adjusting for all baseline characteristics that differed between the participants with and without frequent distressing dreams, that were not included as covariates in the primary analysis. Missing values indicators were used for participants with missing information only on covariates included in the sensitivity analysis, so that all analyses included the same individuals.

Statistical testing was performed two-sided at *P* <0·05. All analyses were performed using SPSS version 28 (IBM Corp., Armonk, NY).

### Ethical considerations

All participants provided written informed consent. Each individual site received institutional review board approval before commencement of the study. The present study received approval from the University of Birmingham (Ref No ERN_21-0427).

### Role of the funding source

There was no funding source for this study. AIO had full access to the dataset and had final responsibility for the decision to submit for publication.

## Results

Amongst 3818 men included in this analysis (mean [SD] age = 77·0 [5·5] years), 368 (9·6%) reported frequent distressing dreams at baseline. Baseline characteristics of the participants stratified by distressing dream frequency are presented in Table 1. The participants with frequent distressing dreams (≥1/week) had lower education, poorer cognitive function, worse physical and mental health, drank less alcohol, were less physically active, and were more likely to use medications that can affect dreaming.

**Table 1:** Baseline characteristics by frequency of distressing dreams (DD).

Characteristic	Non-frequent DD (*n* = 3450)	Frequent DD (*n* = 368)	*P* value
Age (years)	77·0 ± 5·5	77·0 ± 5·1	0·89
Race, *n* (%)			0·24
White	3105 (91·3)	318 (86·4)	
Black	119 (3·4)	19 (5·2)	
Asian	116 (3·4)	14 (3·8)	
Other	67 (1·9)	11 (3·0)	
Mixed	43 (1·2)	6 (1·6)	
Education, *n* (%)			0·002
College degree	1978 (57·3)	193 (52·4)	
High school degree	1311 (38·0)	143 (38·9)	
No qualifications	161 (4·7)	32 (8·7)	
Smoking status, *n* (%)			0·71
Current	66 (1·9)	8 (2·2)	
Past	2008 (58·2)	221 (60·1)	
Never	1376 (39·9)	139 (37·8)	
Alcohol intake, drinks/wk, *n* (%)			0·02
<1	1606 (46·6)	196 (53·3)	
1-13	1643 (47·6)	160 (43·5)	
≥14	201 (5·8)	12 (3·3)	
Diabetes, *n* (%)			0·02
Yes	463 (13·4)	66 (17·9)	
No	2987 (86·6)	302 (18·1)	
Hypertension, *n* (%)			<·001
Yes	1683 (48·8)	225 (61·1)	
No	1767 (51·2)	143 (38·9)	
Self-rated health, *n* (%)			<·001
Good/excellent	3064 (88·8)	302 (82·1)	
Poor/fair	386 (11·2)	66 (17·9)	
Physical activity score^a^	145·0 ± 72·0	136·5 ± 74·3	0·01
Depression, *n* (%)			<·001
Yes	174 (5·0)	47 (12·8)	
No	3276 (95·0)	321 (87·2)	
Anxiety, *n* (%)^b^			<·001
Yes	206 (6·0)	54 (14·7)	
No	2497 (72·4)	244 (66·3)	
Missing	747 (21·7)	70 (19·0)	
Sleep onset insomnia, times/wk, *n* (%)			<·001
0	1822 (52·8)	140 (38·0)	
<1	928 (26·9)	82 (22·3)	
1-2	396 (11·5)	81 (22·0)	
≥3	304 (8·8)	65 (17·7)	
Sleep maintenance insomnia, times/wk, *n* (%)			<·001
0	493 (14·3)	25 (6·8)	
<1	404 (11·7)	22 (6·0)	
1-2	556 (16·1)	44 (12·0)	
≥3	1997 (57·9)	277 (75·3)	
Excessive daytime sleepiness, *n* (%)^c^			<·001
Yes	319 (9·2)	57 (15·5)	
No	2388 (69·2)	242 (65·8)	
Missing	743 (21·5)	69 (18·8)	
Global cognitive function, *n* (%)^d^			<·001
Normal cognition	2270 (65·8)	213 (57·9)	
Mild-severe cognitive impairment	435 (12·6)	86 (23·4)	
Missing	745 (21·6)	69 (18·8)	
Apnoea-hypopnea index, events/hr, *n* (%)^e^			0·16
<15	1377 (39·9)	135 (36·7)	
≥15	1150 (33·3)	141 (38·3)	
Missing	923 (26·8)	92 (25·0)	
Medication use, *n* (%)			<·001
Yes	1616 (46·8)	224 (60·9)	
No	1834 (53·2)	144 (39·1)	

Plus-minus values are means ± SD.

a Physical Activity Scale for the Elderly score. Scores range from 0-793, with higher scores indicating higher physical activity levels.

b Data was missing for 817 participants due to non-completion of the questionnaire.

c Data was missing for 812 participants due to non-completion of the questionnaire.

d Data was missing for 814 participants due to non-completion of the cognitive assessment.

e Measured as number of apnoeas and hypopneas per hour of sleep with a desaturation ≥3%. Data was missing for 1015 participants who did not undergo polysomnography at baseline.

During a total follow-up of 12 years (mean 7·3 years), 91 cases of incident PD (2·4%) were identified. Baseline characteristics of the participants stratified by incident PD status are presented in Table 2. At baseline, the incident PD group were more likely to be depressed and had less frequent sleep maintenance insomnia. There were no other differences in characteristics between the participants that did and did not develop PD.

**Table 2:** Baseline characteristics by incident Parkinson's disease (PD) status.

Characteristic	No PD (*n* = 3727)	Incident PD (*n* = 91)	*P* value
Age (years)	77·0 ± 5·5	76·7 ± 5·4	0·51
Race, *n* (%)			0·41
White	3340 (89·6)	83 (91·2)	
Black	134 (3·6)	4 (4·4)	
Asian	130 (3·5)	0 (0·0)	
Other	76 (2·0)	2 (2·2)	
Mixed	47 (1·3)	2 (2·2)	
Education, *n* (%)			0·60
College degree	2115 (56·7)	56 (61·5)	
High school degree	1424 (38·2)	30 (33·0)	
No qualifications	188 (5·0)	5 (5·5)	
Smoking status, *n* (%)			0·60
Current	72 (1·9)	2 (2·2)	
Past	2180 (58·5)	49 (53·8)	
Never	1475 (39·6)	40 (44·0)	
Alcohol intake, drinks/wk, *n* (%)			0·66
<1	1762 (47·3)	40 (44·0)	
1-13	1756 (47·1)	47 (51·6)	
≥14	209 (5·6)	4 (4·4)	
Diabetes, *n* (%)			0·42
Yes	519 (13·9)	10 (11·0)	
No	3208 (86·1)	81 (89·0)	
Hypertension, *n* (%)			0·34
Yes	1858 (49·9)	50 (54·9)	
No	1869 (50·1)	41 (45·1)	
Self-rated health, *n* (%)			0·80
Good/excellent	3285 (88·1)	81 (89·0)	
Poor/fair	442 (11·9)	10 (11·0)	
Physical activity score^a^	143·9 ± 71·9	154·0 ± 144·2	0·44
Depression, *n* (%)			<·001
Yes	208 (5·6)	13 (14·3)	
No	3519 (94·4)	78 (85·7)	
Anxiety, *n* (%)^b^			0·72
Yes	252 (6·8)	8 (8·8)	
No	2676 (71·8)	65 (71·4)	
Missing	799 (21·4)	18 (19·8)	
Sleep onset insomnia, times/wk, *n* (%)			0·28
0	1909 (51·2)	53 (58·2)	
<1	994 (26·7)	16 (17·6)	
1-2	465 (12·5)	12 (13·2)	
≥3	359 (9·6)	10 (11·0)	
Sleep maintenance insomnia, times/wk, *n* (%)			0·04
0	500 (13·4)	18 (19·8)	
<1	419 (11·2)	7 (7·7)	
1-2	579 (15·5)	21 (23·1)	
≥3	2229 (59·8)	45 (49·5)	
Excessive daytime sleepiness, *n* (%)^c^			0·17
Yes	362 (9·7)	14 (15·4)	
No	2569 (68·9)	61 (67·0)	
Missing	796 (21·4)	16 (17·6)	
Global cognitive function, *n* (%)^d^			0·56
Normal cognition	2419 (64·9)	64 (70·3)	
Mild-severe cognitive impairment	510 (13·7)	11 (12·1)	
Missing	798 (21·4)	16 (17·6)	
Apnoea-hypopnea index, events/hr, *n* (%)^e^			0·15
<15	1467 (39·4)	45 (49·5)	
≥15	1266 (34·0)	25 (27·5)	
Missing	994 (26·7)	21 (23·1)	
Medication use, *n* (%)			0·50
Yes	1793 (48·1)	47 (51·6)	
No	1934 (51·9)	44 (48·4)	

Plus-minus values are means ± SD.

a Physical Activity Scale for the Elderly score. Scores range from 0-793, with higher scores indicating higher physical activity levels.

b Data was missing for 817 participants due to non-completion of the questionnaire.

c Data was missing for 812 participants due to non-completion of the questionnaire.

d Data was missing for 814 participants due to non-completion of the cognitive assessment.

e Measured as number of apnoeas and hypopneas per hour of sleep with a desaturation ≥3%. Data was missing for 1015 participants who did not undergo polysomnography at baseline.

In the fully adjusted logistic regression model (Table 3), compared with participants without frequent distressing dreams at baseline, participants with frequent distressing dreams had a 2-fold risk for incident PD (OR, 2·01; 95% CI, 1·1-3·6, *P* = 0·02).

**Table 3:** Logistic regression models for incident Parkinson's disease by distressing dream frequency at baseline.

Model	PD cases/*n*	OR (95% CI) for Frequent DD vs Non-frequent DD	*P* value
Main analysis			
Age- and Clinic Adjusted Model	91/3818	2·02 (1·2, 3·5)	0·01
Fully Adjusted Model^a^	91/3818	2·01 (1·1, 3·6)	0·02
Stratified analysis^b^			
Baseline to < 5·5 years	30/1438	3·38 (1·3, 8·7)	0·01
5·5 to 12 years	61/2380	1·55 (0·7, 3·3)	0·26
Sensitivity analysis^c^	91/3818	1·98 (1·1, 3·6)	0·02

Abbreviations: PD, Parkinson's disease; OR, odds ratio; CI, confidence interval; DD, distressing dreams.

a Adjusted for age, clinic site, race, education, smoking status, alcohol intake, depression, sleep onset insomnia, sleep maintenance insomnia, diabetes, hypertension, physical activity, self-rated health and medication use.

b Analysis stratified according to length of follow-up, with adjustment for covariates in the fully adjusted model.

c Sensitivity analysis further adjusted for anxiety, cognitive function and excessive daytime sleepiness, in addition to covariates in the fully adjusted model.

In the stratified analysis, which divided the follow-up into two time-periods (Table 3), frequent distressing dreams were associated with a greater than 3-fold risk for incident PD during the first 5 years after baseline (OR, 3·38; 95% CI, 1·3-8·7; *P* = 0·01). However, this association had attenuated and was no longer significant for PD cases that were identified during the subsequent 7 years (OR, 1·55; 95% CI, 0·7-3·3; *P* = 0·26).

In the sensitivity analysis that adjusted for all characteristics that differed between the participants with and without frequent distressing dreams at baseline (Table 3), the association between frequent distressing dreams and incident PD was similar and remained significant (OR, 1·98; 95% CI, 1·1-3·6; *P* = 0·02).

## Discussion

In this large prospective study of community-dwelling older men without PD from the USA, frequent distressing dreams were found to be associated with an increased risk for incident PD during a 12-year follow-up, accounting for a wide range of possible confounders.

This is the first study to investigate the association between dream content in community-dwelling older adults and the subsequent development of PD. Of note, a recent population-based prospective study investigated the association between DEBs (the cardinal symptom of idiopathic RBD [iRBD]) and PD risk, and found that DEBs were associated with a 2·9-fold risk for incident PD during a 3-year follow-up.^23^ Given that iRBD is considered to be the strongest prodromal marker for PD,^4^ the finding of a 3·4-fold risk for incident PD amongst participants with frequent distressing dreams in this study - during an even longer follow-up duration, is a highly significant finding.

The findings of this longitudinal study, are consistent with prior cross-sectional studies, which demonstrated that distressing dreams are very common in people with PD,^5^^,^^6^ and that people with PD are significantly more likely to report having frequent distressing dreams than adults in the general population.^7^^,^^8^ Indeed, studies have shown that 17% of patients with non-demented PD report weekly nightmares,^7^ and this increases to 78% for patients with PD dementia (PDD).^24^ In contrast, estimates for the prevalence of weekly nightmares in the general population range from 2% to 5%.^12^, ^13^, ^14^

An earlier study found that 81% of patients with PD who reported having frequent intense, vivid and frightening dreams, shortly after the time of diagnosis, believed that the changes to their dream content preceded the onset of their motor symptoms by several years, or even decades.^8^ Thus, the findings of the current study provide prospective evidence to support the patients retrospective recall of prodromal distressing dreams. Given that patients with distressing dreams at the time of diagnosis may be five times more-likely to progress from mild PD to moderate-severe PD (Hoehn & Yahr stage ≥ 3) within 5 years,^9^ and may experience cognitive decline at a rate 33-times faster than patients without dream content changes,^9^ it is probable that a significant proportion of the participants with distressing dreams in this cohort, who later developed PD, would have gone on to experience rapid disease progression in the years following their PD diagnosis. As such, this study suggests that screening for distressing dreams in the general population may not only help to identify individuals at risk of developing PD, but may also identify a distinct subtype of PD upon diagnosis, which may help clinicians to provide personalised management for these patients.

In this study, frequent distressing dreams were associated with a greater than 3-fold risk for PD during the first 5 years after baseline, though this association had substantially attenuated during the subsequent 7 years. This suggests that late onset distressing dreams, rather than life-long distressing dreams, may be linked with increased PD risk. Interestingly, population-based cross-sectional studies have shown that nightmares in adulthood are most prevalent from around 70 years of age,^13^^,^^25^ which is strikingly similar to the median age at which PD symptoms first develop.^26^ Moreover, between adolescence and middle adulthood, women are significantly more likely than men to experience weekly nightmares, yet from age 65 onwards, there is no longer a gender gap in nightmares.^13^^,^^25^ This suggests that the age-related increase in dysphoric dreams is most pronounced in men. Thus, these findings indicate that new onset distressing dreams in older adults, particularly older men, may represent early signs of neurodegeneration in some individuals. This hypothesis is in keeping with the findings from the present study, and is consistent with a prior study, which showed that men with PD may have significantly more disturbing dreams than women with PD.^6^ Given that recurrent nightmares are treatable,^27^ future studies are required to determine whether treating distressing dreams may have neuroprotective effects.

The neural correlates of dreaming have only recently begun to be identified.^28^ There is accumulating evidence that the same brain regions involved in regulating emotions during wakefulness, are also involved in regulating emotions during dreaming.^29^^,^^30^ During rapid eye movement (REM) sleep – the sleep stage where nightmares and bad dreams typically occur – activity and blood flow in limbic and paralimbic areas is increased,^5^ which could explain the characteristic hyperemotionality of dreams.^5^ In contrast, activity in frontoparietal associative networks is decreased during REM sleep,^31^ which leads to diminished insight and reduced cognitive control.^5^

A recent study in patients with PD identified that structural changes in the right frontal lobe (right superior frontal gyrus and right anterior cingulate) were correlated with an increased frequency of distressing dreams.^32^ An earlier study identified that patients with left-onset PD (indicating greater right hemisphere pathology) were more likely to have frequent distressing dreams than patients with right-onset PD (indicating greater left hemisphere pathology), and also healthy controls; with the latter two groups showing no difference.^33^ Distressing dreams have also been shown to correlate with poor cognitive function in PD,^34^ particularly frontal executive function,^34^ and also with severity of depression and anxiety.^35^ Given that cognitive impairment and depression usually co-occur in PD, and are each associated with increased PD risk,^4^ it is plausible that distressing dreams and depressive symptoms in the prodromal stage of PD, are both caused by neurodegeneration of right frontal brain regions, that are involved in the downregulation of negative emotions across conscious states.^29^^,^^30^ This theory would be consistent with the neurocognitive model of nightmares.^36^

However, whilst the participants with frequent distressing dreams in this study were indeed more likely to be depressed and to have worse cognitive function (Table 1) - as this theory would predict; after adjusting for these characteristics as covariates, distressing dreams remained significantly associated with incident PD. This suggests that distressing dreams may be a prodromal symptom of PD even in individuals with normal cognition, and who are not depressed. Although, a recent study identified that newly diagnosed patients with PD who reported having recurrent dreams with “aggressive” content (the most frequently reported theme in nightmares),^37^ did not significantly differ at baseline from the patients without frequent aggressive dreams, with respects to depression or cognitive function.^9^ However, after 5 years, the patients who reported having frequent aggressive dreams at baseline, were nearly three times as likely to have developed PDD or mild cognitive impairment, and were both more depressed and more anxious.^9^ Thus, it is possible that distressing dreams in the years preceding a PD diagnosis, may reveal subtle frontal executive deficits, that are not yet detectable by neuropsychological tests or daytime mood disturbances, but may manifest initially as an impaired ability to downregulate negative emotions during REM sleep dreaming.

This study has several strengths, including the prospective design, long follow-up period, use of a well validated questionnaire for assessing habitual sleep disturbances, and the inclusion of a wide range of potential confounders. Furthermore, the participants were not selected based on distressing dream frequency or PD diagnosis. Several limitations also warrant discussion. Following previous published studies,^38^ this study relied on self-reported doctor diagnosis to determine incident PD and therefore may have missed or misclassified some cases. This misclassification could have led to an underestimation of the association. Second, it is possible that including covariates with missing data in the sensitivity analysis might have biased those results. However, the results were similar and remained significant when including only participants with complete covariate data (not shown). Third, the questionnaire item used to assess distressing dreams does not clearly distinguish between frequent bad dreams (i.e., distressing dreams without awakenings) and frequent nightmares (i.e., distressing dreams with awakenings). As such, it is not possible to determine whether the associations with PD may vary by distressing dream subtype. Fourth, the relatively small number of incident PD cases weakens the evidence and precluded the evaluation of more precise temporal associations. Finally, the findings from this study might not be generalisable to women and younger adults.

This study provides evidence for the first time that frequent distressing dreams in community-dwelling older adults, may be associated with an increased risk for developing PD. Participants with frequent distressing dreams were nearly three times more likely to develop PD within the initial 5 years of follow-up, however this association had attenuated and was no longer significant during the subsequent 7 years. This suggests that frequent distressing dreams may be a prodromal symptom of PD. As such, screening for late onset distressing dreams in the general population, may help to identify individuals at increased risk of developing a rapidly progressing subtype of PD,^9^ in whom early interventions could be targeted.

## Contributors

AIO was responsible for conception, organisation, and execution of the research project; design and execution of the statistical analysis; verification of the underlying data; and manuscript preparation. AIO had full access to all the data in the study and accepts responsibility for the decision to submit for publication.

## Data sharing

Data from MrOS are available at https://mrosonline.ucsf.edu. The analysis dataset for this specific manuscript are also available from the corresponding author upon request.

## Declaration of Interests

The author declares no conflict of interest.
